# Fever after intraventricular neuroendoscopic procedures in children

**DOI:** 10.1007/s00381-016-3085-3

**Published:** 2016-04-14

**Authors:** S. L. de Kunder, M. P. ter Laak - Poort, J. Nicolai, J. S. H. Vles, E. M. J. Cornips

**Affiliations:** Department of Neurosurgery, Maastricht University Medical Center, Maastricht, The Netherlands; Department of Child Neurology, Maastricht University Medical Center, Maastricht, The Netherlands

**Keywords:** Children, Complications, Endoscopic third ventriculostomy, Neuroendoscopy, Postoperative fever

## Abstract

**Purpose:**

The purpose of this paper was to study the incidence and clinical significance of fever after intraventricular neuroendoscopic procedures in children.

**Methods:**

We retrospectively assessed all children subjected to an intraventricular neuroendoscopic procedure between 2004 and 2015. Body temperature 6 days postoperatively, symptoms and signs, and eventual cerebrospinal fluid analysis were evaluated. Fever was defined as temperature above 38 °C.

**Results:**

Fifty-five children (mean age 4.8 years) had 67 procedures. Forty-three children (47 procedures, 70 %) developed fever, mostly the day of surgery (*n* = 17; 25 %) or the next day (*n* = 33; 49 %). All children who were clinically ill (*n* = 9, including 7 with fever) suffered serious illness, as opposed to none of the children with fever without being clinically ill (*n* = 36). Fever was unrelated to gender, indication for, and type of procedure and did not influence ETV success rate at 3 months. Children under 1 year less frequently developed fever (*p* = 0.032).

**Conclusions:**

Fever frequently develops after intraventricular neuroendoscopic procedures in children and follows a rather predictable course, peaking the day of surgery and/or the next day, and rapidly subsiding thereafter. Fever is not a cardinal symptom except when combined with other symptoms in children who are clinically ill (which most of them are not). Close observation avoiding invasive diagnostic tests may suffice for those who are not clinically ill, while extra attention should be paid to those whose temperature rises after day 2 especially when clinically ill, as they likely suffer serious illness. We recommend to closely observe children after any intraventricular neuroendoscopic procedure for at least 5 days.

## Introduction

Neuroendoscopic procedures have proven to be a reliable and safe alternative to open procedures [1–3]. Fever after intraventricular neuroendoscopic procedures is frequently observed, but not extensively discussed in the literature. Postoperative fever always causes a dilemma for the attending physician who has to decide whether (mostly invasive) diagnostic tests are warranted to determine the cause of the fever, or whether watchful waiting is an option. Of note, young children with chronic medical problems who are subjected to invasive procedures are at greater risk for complications such as dehydration and occult bacteremia as compared to adults [4]. In addition, they are more prone to an imbalance between heat production and heat dissipation, and therefore more vulnerable. For these reasons, the question if and when postoperative fever points to a serious illness is even more relevant in (young) children than in adults. According to a recent paper by Kinoshita et al. [5], the incidence of fever after intraventricular neuroendoscopic procedures in children under 10 years of age may be as high as 84.4 %. The purpose of this paper was to study the incidence and clinical significance of fever after intraventricular neuroendoscopic procedures in children, hoping a better understanding of this phenomenon will help the attending physicians to avoid unnecessary and especially invasive diagnostic procedures.

## Material and methods

This retrospective research is not subject to the Medical Research Involving Human Subjects Act in the Netherlands (WMO in Dutch). All children (less than 18 years old at time of surgery) subjected to an intraventricular neuroendoscopic procedure in our institute between January 2004 and February 2015 were retrospectively analyzed. Children with fever or signs of infection prior to surgery were excluded. All data were obtained from medical records, including such surgical details as the exact kind of procedure, duration of surgery, irrigation fluid used, and eventual complications. Body temperature was measured three times a day (morning, afternoon, and evening) using a tympanic membrane thermometer for at least 6 days postoperatively (the minimum stay according to local protocol). Children with fever (defined as a temperature above 38 °C) were assigned to two categories: those with a temperature in between 38 and 39 °C (mild fever) and those with a temperature above 39 °C (high fever). Clinical symptoms and signs were collected from all children with fever in order to find out whether or not they were clinically ill. ETV success rate was assessed 3 months postoperatively.

### Surgical technique

All procedures were performed under general anesthesia using a peel-away sheath and stylet to puncture the ventricle. The scope used was either a rigid fiberscope (Clarus scope, Medtronic, Minneapolis, USA) or a rod-lens scope (Minop scope, B. Braun - Aesculap, Melsungen, Germany). Ringer’s lactate solution at body temperature was invariably used as irrigation fluid. Perioperative prophylactic antibiotics were administered to all children (flucloxacillin/rifampicin in those <6 months old and cefazolin in those >6 months old).

### Statistical analysis

All data were processed and analyzed with the Statistical Package for the Social Sciences (IBM SPSS Statistics, version 22). Data were tested for normal distribution using a Kolmogorov-Smirnov test and a Shapiro-Wilk test, while a Student’s *t* test was used to compare means, and a chi-square test to compare percentages. Results were expressed as means and standard deviation or percentages. A *p* value <0.05 was considered statistically significant.

## Results

### Patient characteristics and surgical details

**Fig. 1:**
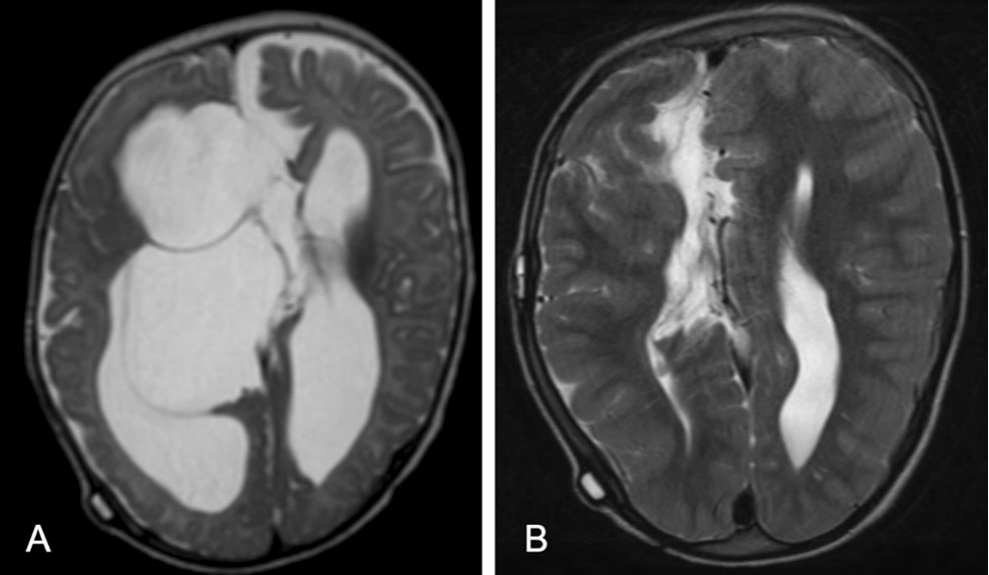
Pre- (**a**) and postoperative (**b**) axial T2-weighted MR images of a child with posthemorrhagic cystic ventricular dilatation and no longer functional ventriculoperitoneal shunt

**Fig. 2:**
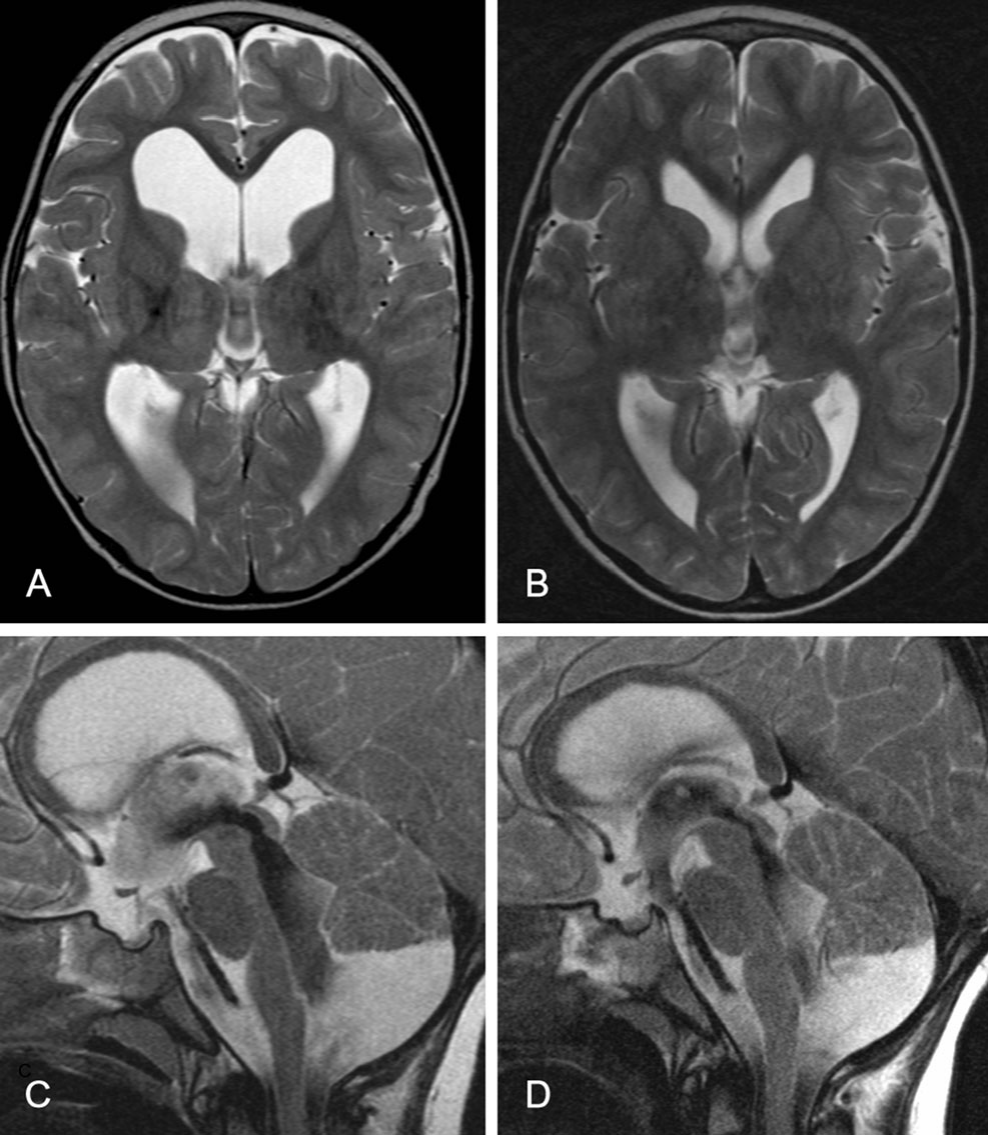
Preoperative axial (**a**) and sagittal (**c**) T2-weighted MR images of a child with Blake’s pouch cyst. Axial (**b**) and sagittal (**d**) T2-weighted MR images after ETV

Table 1 summarizes patient characteristics and surgical details. A total of 62 children had a total of 76 procedures, of which 9 procedures were excluded because of insufficient data due to the retrospective character of the study. The remaining 67 procedures were performed in predominantly male subjects (66 % male vs. 34 % female) with an average age of 4.8 years (0–15; sd 5.4). Indications for surgery were aqueductal stenosis, 4th ventricular outflow problem including Blake’s pouch cyst, Dandy-Walker malformation, arachnoid cyst, intra-axial tumor, or other (Figs. 1 and 2).

**Table 1:** Patient characteristics and surgical details

Number of children	55
Number of procedures	67
Gender	
Male	44 (66 %)
Female	23 (34 %)
Age (years)	4.8 (0–15, sd 5.4)
Indication for surgery	
Aqueductal stenosis	32 (48 %)
4th ventricular outflow problem/Blake’s pouch cyst	12 (18 %)
Dandy Walker malformation	3 (5 %)
Arachnoid cyst	7 (10 %)
Tumor	12 (18 %)
Other	1 (1 %)
Kind of procedure	
ETV^a^	54 (81 %)
ETV and tumor biopsy	7 (10 %)
ETV and cyst puncture	6 (9 %)
Duration of surgery (min)	58 (16–150, sd 27)

^a^Including re-ETV

Table 2 summarizes ETV success rate at 3 months, divided into two age groups (those under 1 year, and those above 1 year) as it has been well established that ETV success rate is substantially lower in children under 1 year [2]. All children had ETV or re-ETV, in most cases (*n* = 54; 81 %) as an isolated procedure. In few cases, ETV was combined with cyst puncture (*n* = 6; 9 %) or tumor biopsy (*n* = 7; 10 %). Average duration of surgery was 58 minutes (16–150; sd 27). In this series, we encountered one major complication, a severe intraventricular hemorrhage in a child who subsequently developed permanent hypothalamic dysfunction. There was no mortality associated with the neuroendoscopic procedures.

**Table 2:** ETV success rate at 3 months

	Under 1 year	Above 1 year	Overall
ETV success rate (3 months)	52 %	71 %	61 %

### Postoperative fever

**Fig. 3:**
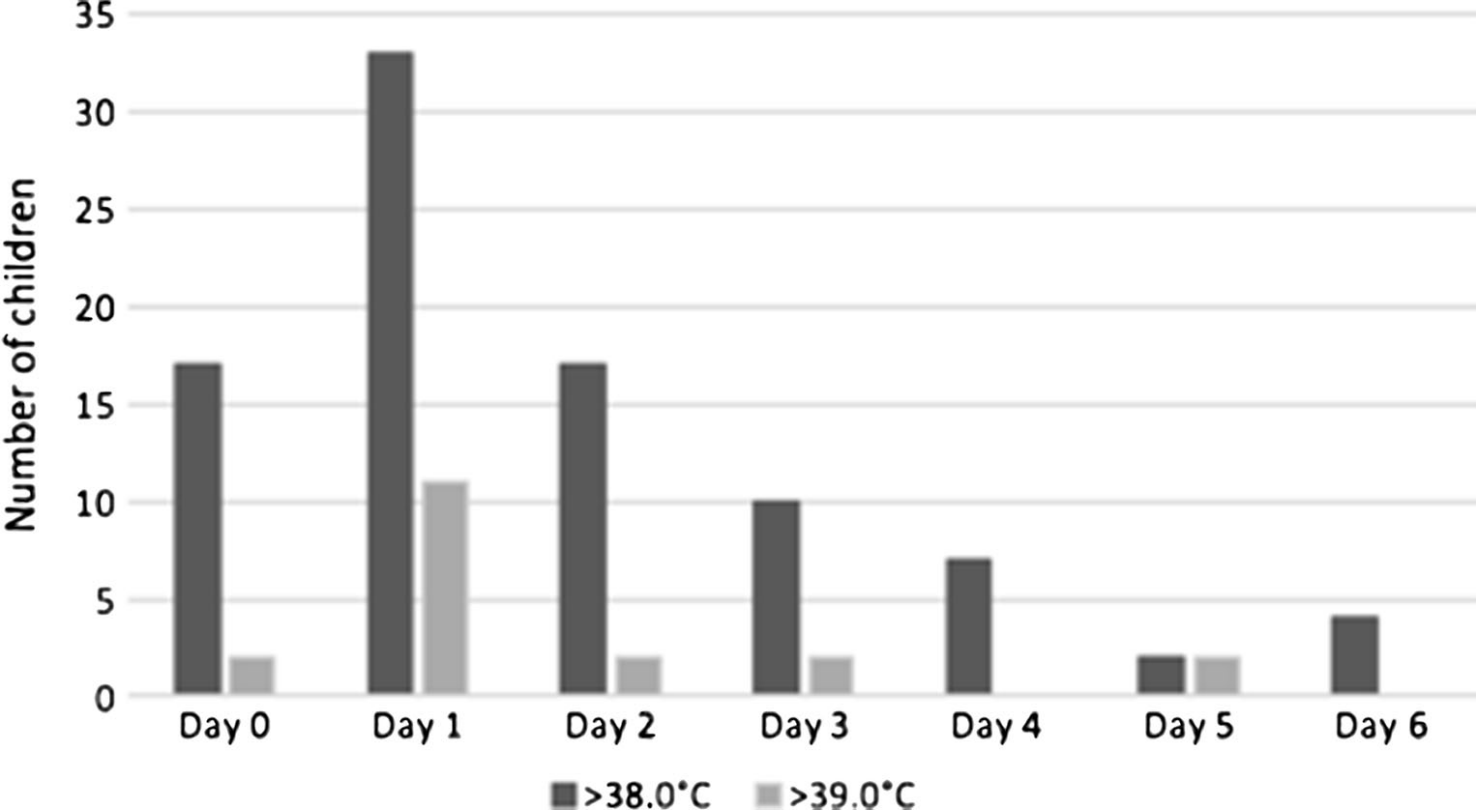
Incidence of postoperative fever

Details are summarized in Table 3. During the specified follow-up period (6 days postoperatively), 43 children (47 (70 %) of all neuroendoscopic procedures) developed a temperature above 38.0 °C, and 15 of them (22 % of all neuroendoscopic procedures) developed a temperature above 39.0 °C. Most children developed fever quite early, more specifically the day of surgery (day 0) or the next day (day 1). In the former group, 17 children (25 % of all neuroendoscopic procedures) had a temperature above 38.0 °C, including 2 (3 %) above 39.0 °C, while in the latter group, 33 children (49 % of all neuroendoscopic procedures) had a temperature above 38.0 °C, including 11 (16 %) above 39.0 °C (Fig. 3). Merely 9 children (13 % of 67 procedures) were clinically ill according to the attending physician’s assessment, most of them having a fever (*n* = 7; 78 %), including 3 with a temperature between 38.0 and 39.0 °C, and 4 with a temperature above 39.0 °C (Table 4).

**Table 3:** Postoperative fever (67 procedures in 55 children)

	>38.0 °C*	>39.0 °C
Day 0	17 (25 %)	2 (3 %)
Day 1	33 (49 %)	11 (16 %)
Day 2	17 (25 %)	2 (3 %)
Day 3	10 (15 %)	2 (3 %)
Day 4	7 (10 %)	0
Day 5	2 (3 %)	2 (3 %)
Day 6	4 (6 %)	0

*Including those with fever >39.0 °C

**Table 4:** Clinically ill children

	No fever	>38.0 °C	>39.0 °C	Underlying condition
Child no. 1		•	•	Meningitis
Child no. 2		•	•	Meningitis
Child no. 3		•		Acute hydrocephalus^a^
Child no. 4		•		Acute hydrocephalus^a^
Child no. 5		•		Tumor progression
Child no. 6		•	•	Gastroenteritis
Child no. 7		•	•	Intraventricular hemorrhage
Child no. 8	•			Acute hydrocephalus^a^
Child no. 9	•			Acute hydrocephalus^a^

^a^Indicating early ETV failure

### Clinically ill children

Nine children were observed to be clinically ill, whereas the remaining ones were fine. More specifically, 9 out of 67 procedures (13 %) were followed by an episode of clinical illness. Four children demonstrated a lowered level of consciousness within 48 hours of the neuroendoscopic procedure. All of them were diagnosed with an acute hydrocephalus (early ETV failure) requiring re-ETV, external ventricular drainage, or internal shunting. Four other children demonstrated nuchal rigidity. One of them previously treated for a thalamic anaplastic astrocytoma was suffering dramatic tumor progression and died within 1 week after the procedure. The other three were suspect for postoperative meningitis and therefore subjected to a lumbar puncture. In two of them, cerebrospinal fluid (CSF) analysis was consistent with bacterial meningitis (low glucose and elevated protein levels); however, in merely one of them, the gram stain was positive and actual bacteria (Pneumococcus) were cultured, while the other one may have had aseptic meningitis, or CSF cultures may have been sterilized due to the perioperative prophylactic antibiotics. Based on their clinical presentation and abnormal CSF analysis, however, both children had broad-spectrum antibiotics for possible bacterial meningitis for a period of 12 days. The third child with reassuring CSF analysis (normal glucose and protein levels) was subjected to an MRI scan because of persisting illness, demonstrating an intraventricular hemorrhage as the likely cause for its symptoms. Another child had symptoms consistent with gastroenteritis and was treated accordingly (without antibiotics). Interestingly, while many children (70 %) had fever on day 0 and/or day 1 after the neuroendoscopic procedure, those who went on to develop an infection (either a meningitis or a gastroenteritis) demonstrated a secondary rise in temperature after day 2. Importantly, we did not observe meningitis or any other infectious disease in any of the children with fever (even above 39 °C) who were not clinically ill.

### Variables predictive for postoperative fever

There was no significant relation between postoperative fever and gender, indication for, and type of intraventricular neuroendoscopic procedure. Moreover, postoperative fever did not seem to influence ETV success rate at 3 months. However, we observed a relation with age, as children under 1 year (*n* = 27; 67 %) were less likely to develop postoperative fever (*p* = 0.032). Moreover, we observed a trend towards significance for duration of surgery, as children with a procedure under 58 minutes (the mean duration of surgery in this series) were more likely to develop postoperative fever (*p* = 0.056) (Table 5). Temperature rising above 38.0 °C 3 to 6 days postoperatively more frequently occurred in clinically ill children (5 out of 9 children, 56 %) as compared to non-clinically ill children (9 out of 58 children, 16 %) and was usually indicative for a serious illness.

**Table 5:** Variables predictive for postoperative fever

	No fever (*n* = 20)	Fever (*n* = 47)	*p* value
Gender			
Male	14	30	0.633
Female	6	17	
Age (years)			0.032
Under 1 year	12	15	
Above 1 year	8	32	
Indication for surgery			0.941
Aqueductal stenosis	11	21	
4th ventricular outflow problem/Blake’s pouch cyst	1	11	
Dandy Walker malformation	2	1	
Arachnoid cyst	2	5	
Tumor	4	8	
Other	0	1	
Kind of procedure			0.694
ETV^a^	17	37	
ETV and tumor biopsy	2	5	
ETV and cyst puncture	1	5	
Duration of surgery (min)			0.056
Under 58′	8	31	
Above 58′	12	16	
ETV success rate (3 months)	10	31	0.156

^a^Including re-ETV

## Discussion

Fever (defined as a temperature above 38.0 °C) after intraventricular neuroendoscopic procedures in both pediatric and adult patients is likely underreported. In several papers on intraventricular neuroendoscopy, transient fever is merely mentioned as frequently occurring postoperatively, but not discussed in any detail [6–9]. Kinoshita et al. [5] recently reported an incidence of 65.1 % in a mixed pediatric and adult patient series, including an incidence of 84.4 % in children under 10 years, whereas we observed an incidence of 70 % (47 out of 67 procedures) in an exclusively pediatric patient series (*n* = 62). Although postoperative fever (even above 39.0 °C) does not seem to influence ETV success rate at 3 months, it is important to better understand the phenomenon. Hence, unnecessary and especially invasive diagnostic procedures may be avoided, and parents, pediatricians, and other caregivers may be reassured, even when the exact pathophysiological mechanism has not yet been elucidated [10].

Body temperature is the result of a delicate balance between heat production and heat dissipation [11], coordinated by a complex system involving temperature regulating centers situated in the hypothalamus. A large number of heat- and cold-sensitive neurons in the preoptic nucleus of the anterior hypothalamus function as temperature sensors. Whenever the preoptic nucleus is heated, the body reacts with profuse sweating and excess body heat production is inhibited. At the posterior hypothalamus, peripheral thermal input from the skin is processed and combined with signals from the preoptic nucleus of the anterior hypothalamus to maintain a balanced body temperature [12, 13]. In this regard, Chernov et al. [10] postulated that fever after intraventricular neuroendoscopic procedures may be the result of irritation of the hypothalamus due to mechanical stimulation (while perforating the third ventricular floor), small hemorrhages, and/or air in the ventricular system. On a cellular level, cytokines play an important role in the development of fever. Several animal studies have shown that the direct injection of certain cytokines, especially interleukin-1 (IL-1) and prostaglandin E_2_, in the hypothalamus results in an immediate or delayed temperature rise [13, 14]. Of note, children are different from adults as their blood-brain barrier may still be immature and their developing brain is not the strictly controlled, stable environment an adult brain is [15]. We hypothesize an intraventricular neuroendoscopic procedure may easily cause a disturbance in this vulnerable environment contributing to the development of fever.

Fever was most frequently observed the day of surgery (*n* = 17; 25 %) or the next day (*n* = 33; 49 %) in our patient series. In this early postoperative period, four patients demonstrated a lowered level of consciousness, and all of them were diagnosed with an acute hydrocephalus (early ETV failure) requiring another neurosurgical intervention. As merely two of them (50 %) had a fever, which is less than the overall incidence in our patient series (*n* = 47; 70 %), early postoperative fever is not indicative for early ETV failure. Likewise, postoperative fever (even above 39.0 °C) does not seem to influence ETV success rate at 3 months (61 % overall ETV success rate, 66 % in those with and 50 % in those without postoperative fever, the latter group including 60 % children under 1 year) (Table 5). Most of these children with fever on day 0 and/or day 1 had a rapid subsidence within the next few days and an otherwise uneventful postoperative course (Fig. 3).

Fever was less frequently observed more than 2 days postoperatively (*n* = 14; 21 % of 67 procedures). Five children (36 %) were clinically ill, and all of them were soon diagnosed with a serious illness, including meningitis (one bacterial, one possibly aseptic), gastroenteritis, tumor progression, or intraventricular hemorrhage (one child each). Nine children (64 %) were not clinically ill, and all of them had an otherwise uneventful postoperative course.

Comparing our series to the series recently reported by Kinoshita et al. [5], we observe some similarities as well as some differences. Of note, these authors present a series of both adults and children with a mean age of 25.7 (sd 24.0) and a median age of 14.0 years (range 0.2–86.3), whereas we present an exclusively pediatric series. Dividing their series in two age groups, the incidence of postoperative fever in patients under 10 years was 84.4 %, and in those above it was 52.8 %. In our series, with a mean age of 4.8 years (0–15; sd 5.4), the overall incidence of postoperative fever was 70 % (*n* = 47). More specifically, the incidence was 56 % in children under 1 year and 80 % in children above 1 year (Table 5). Of note, the latter incidence is almost identical to the one reported by Kinoshita et al. [5]. Other differences between both series involve the duration of surgery and the irrigation fluid used. Whereas our average duration of surgery was 58 minutes (median 50 minutes), their average duration of surgery was 105.5 minutes (median 102 minutes). Interestingly, while in our series a shorter procedure seems to correlate with an increased incidence of postoperative fever (30/38 under 58′ versus 16/28 above 58′, *p* = 0.056, Table 5), the substantial difference in the duration of surgery between our series and the Kinoshita series (almost twice as long) does not translate in a substantially different incidence of postoperative fever. We therefore postulate that duration of surgery is not a major determinant for the occurrence of fever after intraventricular neuroendoscopic procedures. Finally, in our hospital irrigation fluids are always pre-heated to body temperature (37.0–38.0 °C), whereas in the Kinoshita series they were at room temperature (personal communication). We again postulate that irrigation fluid temperature is not a major determinant for the occurrence of fever after intraventricular neuroendoscopic procedures. Finally, all children in the present series had an ETV with or without additional procedures such as cyst puncture or tumor biopsy, whereas in the Kinoshita series 17 patients (21 %) did not have an ETV. Therefore, at least in our series, we cannot exclude ETV was the sole responsible for the development of fever that may not develop in other intraventricular neuroendoscopic procedures without ETV.

## Conclusion

To the best of our knowledge, we present the first exclusively pediatric patient series on fever following intraventricular neuroendoscopic procedures. Although we believe the quality of the data in this retrospective study is good (after elimination of 9 procedures because of insufficient data), it remains difficult to draw solid conclusions with regard to the factors that may influence fever occurring postoperatively. However, from this study, we learn that fever frequently develops after intraventricular neuroendoscopic procedures in children, especially those above 1 year of age. It follows a rather predictable course, peaking the day of surgery and/or the next day and rapidly subsiding usually within 72 hours. Moreover, these patients are typically not clinically ill. Therefore, fever by itself is not a cardinal symptom the first few days postoperatively, except when combined with other symptoms and signs in children who are clearly clinically ill. Close observation may suffice for those who are not clinically ill, whereas extra attention should be paid to those whose temperature rises after day 2 especially when clinically ill, as they likely suffer a serious illness. We hope a better understanding of the phenomenon of fever after intraventricular neuroendoscopic procedures will help the attending physicians to avoid unnecessary and especially invasive diagnostic procedures. We recommend to closely observe children after any intraventricular neuroendoscopic procedure for at least 5 days.

## Abbreviations

C: Celsius
CSF: Cerebrospinal fluid
ETV: Endoscopic third ventriculostomy
